# Health professional’s readiness and factors associated with telemedicine implementation and use in selected health facilities in Ghana

**DOI:** 10.1016/j.heliyon.2023.e14501

**Published:** 2023-03-17

**Authors:** Nathan Kumasenu Mensah, Godwin Adzakpah, Jonathan Kissi, Richard Okyere Boadu, Obed Uwumbornyi Lasim, Martha Khainde Oyenike, Abigail Bart-Plange, Maxwell Ayindenaba Dalaba, Felix Sukums

**Affiliations:** a Department of Health Information Management, School of Allied Health Science, University of Cape Coast, Cape Coast, Ghana; b Institute of Health Research, University of Health and Allied Sciences, Ho, Ghana; c Muhimbili University of Health and Allied Sciences, Box 65001, Dar es Salaam, Tanzania

**Keywords:** Telemedicine, Low- and middle- income countries (LMICs), eHealth implementation, Health professionals, Readiness, Ghana

## Abstract

**Background:**

Telemedicine, which is the practice of medicine using technology to deliver health care remotely, has a low adoption rate in low- and middle-income countries (LMICs). However, the advent of coronavirus disease 2019 (COVID-19) has forced healthcare systems in these settings to begin implementing telemedicine programs. It is unknown how prepared health professionals and the healthcare system are to adopt this technology. Therefore, this study aimed to assess the readiness of health professionals and explore factors associated with telemedicine implementation in Ghana.

**Methods:**

A cross-sectional study was conducted in six health facilities between March and August 2021. Convenience sampling was used to select the six health facilities, and the participants were selected randomly for the study. Questionnaires were self-completed by participants. Data was exported into STATA 15.0 for analysis, and appropriate statistical methods were employed. All statistical tests were performed at a significance level of p < 0.05.

**Results:**

Of the 613 health professionals involved in the study, about 579 (94.5%) were comfortable using computers, and the majority, 503 (82.1%) of them, had access to computers at the workplace. Health professionals agreed that the measures outlined by the health facilities supported their readiness to use telemedicine for healthcare services. Analysis revealed a statistically significant positive relationship between health facilities’ core readiness and health professionals’ readiness, with a correlation coefficient (r) of 0.5484 and a p-value<0.0001. Of the factors associated with health professionals’ readiness towards telemedicine implementation, facility core readiness, engagement readiness, staff knowledge and attitude readiness showed a statistically significant relationship with health professionals’ readiness.

**Conclusion:**

The study revealed that health professionals are ready to adopt telemedicine. There was a statistically significant relationship between health facilities’ core readiness, engagement readiness, staff knowledge and attitude readiness, and health professionals’ readiness. The study identified factors facilitating telemedicine adoption.

## List of abbreviations

CCTH: Cape Coast Teaching Hospital
COVID-19: Coronavirus disease 2019
EMR: Electronic Medical Record
ICT: Information Communication Technologies
KMO: Kaiser–Meyer–Olkin
LMICs: Low- and Medium-Income Countries
MSA: Measure of Sampling Adequacy
UGMC: University of Ghana Medical Centre

## 1 Introduction

There has been considerable advocacy towards exploiting electronic health (eHealth) services' potential to enhance healthcare quality and safety [1]. With the advent of the Coronavirus disease 2019 (COVID-19), this advocacy has become more relevant, and healthcare systems have begun to use more eHealth services due to the imposition of social isolation restrictions [2,3]. Also, innovations and technological advancements have made internet communication cheaper, providing a unique opportunity for integrating telemedicine into healthcare practices, especially in low- and middle-income countries (LMICs) [[4], [5], [6]]. However, many LMICs face challenges, such as lack of an appropriate policy framework to guide how telemedicine should be successfully implemented [7,8], especially in settings where acceptability and adoption rates of similar technologies have been notoriously low [1,9]. The health needs and challenges in many LMICs are also different [10]. Several persons living in rural areas in most LMICs have challenges accessing high-quality healthcare [11].

In Ghana, the mode of primary health care delivery is rooted in Community-based health planning and services (CHPS), health posts and health centres. This method of conventional primary health services delivery comes with several challenges. As a result of inadequate funding sources, health facilities struggle to employ adequately trained health professionals to man these facilities amid the high volume of patients in need of care. According to the World Bank, about 42% of Ghana’s population in 2021 live in rural areas [12]. Unfortunately, this section of the population is the most disadvantaged by the unequal allocation of healthcare resources [11]. In 2017, Ghana’s doctor-to-population ratio was 1:7374 and nurse to population ratio was 1:505 [13]. These ratios are even more prevalent in rural healthcare delivery.

Also, the distribution of health infrastructures such as pharmacies, clinics, and hospitals across the country is concentrated in urban areas, often with fewer or no such health facilities in the rural areas. Due to these challenges, patients are more likely to travel long distances to access improved healthcare services in urban areas [14]. Additionally, the majority of specialist physicians are stationed in urban health facilities. This means that cases that cannot be handled by doctors in rural areas must be referred to urban health facilities.

There is a need for a system that will reduce costs while ensuring that patients receive satisfactory healthcare irrespective of their geographical locations. Telemedicine offers solution to these issues as a result of rapid developments in Information and Communication Technologies (ICT). Unfortunately, large-scale investments in ICT infrastructure across Africa have been inadequate, in part due to inadequate or lack of funding in some regions. In addition, the likelihood of providing telemedicine is extremely low in rural areas, where it will be most beneficial to the poor because of the enormous infrastructure requirements and connectivity costs [15]. Outside of the urban area, internet connectivity is also poor [16].

Telemedicine has been defined as using computers and related accessories such as cameras, speakers, high-speed internet and other ICT tools to administer and facilitate healthcare delivery over distance as an alternative to face-to-face interactions between clinicians and patients [17,18].

Telemedicine has a remote capability that has been exploited to offer speciality care, for rapid assessment and treatments. In high-income countries, it has been used to address health issues such as the health disparity between health seekers’ needs and health service availability [19], to improve obstetric care outcomes [5,20] and to provide solutions to issues of shortage of health professionals, especially specialist in underserved communities [6]. With the ageing population and age-related chronic diseases increasing worldwide, telemedicine has been an avenue for accessing healthcare among the aged and to reduced frequent visits to health facilities [6]. Evidence shows that the use of telemedicine for home healthcare services has resulted in reduced mortality, better medication compliance and improved safety [21], albeit, most of these studies were conducted in high-income countries. The occurrence of the COVID-19 pandemic has demonstrated that an immense burden can be placed upon both health professionals and health facilities. Hospitals have been forced to make rapid preparation towards transitioning to telemedicine to alleviate this burden [5]. According to Scott and others, telemedicine can improve the capabilities of health professionals while also reducing the likelihood of crowd contact and virus transmission [22]. Kronfield and colleagues stated that telemedicine services played a crucial role in halting the progression of adverse diseases during any pandemic and that health information technology provides us with a more suitable means of monitoring cancers and chronic diseases [23]. The concept of telemedicine enables healthcare professionals to enrol in e-learning programs to build their continuous professional education [24].

The transition towards telemedicine is rapidly growing globally, however, only a few telemedicine projects initiated in LMICs have reached maturity level. The slow uptake of telemedicine in many LMICs relates to factors such as inadequate infrastructure, slow acceptance, inadequate technological equipment, limited financial resources, and inadequate skilled human resources [[25], [26], [27], [28], [29]]. Other reasons accounting for the slow uptake of LMICs are failure to assess health facility readiness before the implementation [30]. Studies have shown that the availability of telemedicine is not sufficient condition for its usage [31] but depends on quality-oriented culture, the self-sufficiency of the hospital, and how flexible the hospital functions [32]. The readiness to implement and use telemedicine may vary with the type of health facility [31,32]. Therefore, health facilities and professionals’ readiness must be assessed before the implementation of telemedicine [7,8,18,[33], [34], [35]]. This study assessed the readiness of health professionals and explored factors associated with the implementation and use of telemedicine in six selected study sites in Ghana.

## 2 Methods

### 2.1 Study design

An institutional-based cross-sectional study was carried out in six selected health facilities in Ghana between March and August 2021. All six health facilities were selected on the basis that they used an Electronic Medical Record system (EMR) to provide care, which could serve as a proxy for integrating telemedicine services.

### 2.2 Study area

The study was conducted in six purposively selected health facilities in Ghana. Two tertiary facilities, namely; the University of Ghana Medical Centre (UGMC) and Cape Coast Teaching Hospital (CCTH), and four other secondary health facilities, namely; Essikado District Hospital, Eastern Regional Hospital, Baptist Medical Centre and Mab International Hospital.

### 2.3 Study population

The study targeted health professionals employed in the selected health facilities. They comprised of nurses/midwives, physicians, pharmacists, radiologists/sonographers, physiotherapists, and dietetics/nutritionists.

### 2.4 Sample size, sampling and sampling procedure

The minimum sample size for this study was based on a priori power calculation [36]. Thus, we sampled a minimum of 194 respondents, as this would provide enough statistical power (0.80) to detect small-sized correlation coefficients (0.20) [36]. This made room for a larger sample size, as it would increase the statistical power for detecting smaller eﬀects and strengthen the robustness of the findings. Convenience sampling was used to select respondents who were available and willing to participate in the study. Table 1 below shows the distribution of selected respondents by health facility.

**Table 1:** Distribution of sample by health facility.

Name of Health facility	Selected sample	Per cent
Baptist Medical Centre, Nalerugu	150	24.47
Cape Coast Teaching Hospital, Cape Coast	99	16.15
Eastern Regional Hospital, Koforidua	117	19.09
Essikado District Hospital, Essikado	86	14.03
Mab International Hospital, Accra	63	10.28
University Ghana Medical Centre, Accra	98	15.99
Total	613	100

### 2.5 Operational definitions

*Core readiness* was defined as realising the need for telemedicine and having an appropriate plan for its implementation drawn by policymakers and user groups, which includes budget and identifying resources needed to integrate telemedicine with current services.

*Engagement readiness* was defined as correctly identifying prioritized needs in the facility that telemedicine will address and express dissatisfaction with the current way of working and awareness of the role of telemedicine among staff.

*Knowledge and attitude* of health professionals’ readiness were defined as having the proper knowledge and positive attitude towards telemedicine.

*Health professionals' readiness* was defined as staff's general comfort and willingness to use telemedicine [37].

### 2.6 Inclusion and exclusion criteria

All 613 health professionals working in the six selected facilities and participating in the telemedicine survey were included in the study for higher precision and accuracy. These health professionals include nurses/midwives, physicians, pharmacists, radiologists/sonographers, physiotherapists, and dietetics/nutritionists. Excluded from the study were health professionals who were less than six months in their current position. Health professionals such as health information officers and laboratory staff were also excluded from the survey.

### 2.7 Data collection instruments and procedures

Health professionals' responses on their readiness for Telemedicine adoption were gathered using a structured and pretested self-administered questionnaire modified from Ref. [38] “readiness assessment instrument”. Other resource-constrained contexts have validated and used this tool [39,40]. In a South African study, the Cronbach alpha was 0.864 and 0.910 for core and engagement readiness [41]. This is over the cut-off of 0.7, suggesting good instrument reliability. Since the instrument was created initially to gauge provider and patient enthusiasm for the adoption of eHealth, the original device was modified to fit the purpose of this study Health professionals’ readiness and factors associated with telemedicine implementation and use. The questionnaires were distributed to respondents who were required to self-complete them. The questionnaires were collected later so as not to disrupt the working schedules of the survey participants. Six data collectors and two supervisors participated in the data collection. A data collector was stationed in each facility, and each supervisor was responsible for three facilities. A one-day training was provided to the data collectors and the supervisors. The training focused on the objectives of the study and the data collection process.

The survey questionnaire had a total of 31 questions in 5 thematic sections aiding the objectives of the research. Section A comprised socio-demographic; section B was on the core readiness toward implementation of telemedicine systems; section C comprised engagement readiness toward implementation of telemedicine; section D was on behavioural (Knowledge and Attitude) of health professionals' readiness towards implementation of the telemedicine system, and Section E assessed health professionals’ readiness towards implementation of telemedicine systems at the facility.

### 2.8 Data processing and management

All the questionnaires were checked for accuracy, completeness and legibility before being entered into an electronic data-capturing tool developed using EpiData 3.1 software. The data screens had in-build checks to minimize data entry errors. Each completed questionnaire was assigned a unique number for quality control and easy recall purposes.

### 2.9 Data analysis

The data was exported and converted into STATA Version 15 for analysis. Descriptive statistics (such as frequencies, means and standard deviations) were used to describe the data. To achieve the test of normality, Shapiro-Wilk and Bartlett's tests were used to assess the symmetry of all continuous data. The reliability of the variables in the datasets was evaluated by examining Cronbach’s alpha coefficient. The measure of sampling adequacy (MSA) using Kaiser–Meyer–Olkin (KMO) test and Bartlett's test of sphericity were also calculated. The KMO overall measure of sampling adequacy (MSA) was 0.910, which falls within the acceptable level, and was significant at p < 0.0001. The Bartlett's test of sphericity (degree of freedom = 465) was 8839.944 and p-value<0.0001, indicating a highly significant correlation among the survey questions. Participants' readiness was assessed by calculating the overall readiness score for each respondent. Spearman’s rank correlation coefficient was performed to assess the relationship between health professionals' readiness and other readiness factors. Unadjusted regression was conducted to explore factors influencing health professionals' readiness for telemedicine implementation. All statistical tests were performed at a significance level of *p* < 0.05.

### 2.10 Ethical approval

The study received ethical approval from the University Cape Coast Institutional Review Board (UCCIRB/CHAS/2021/62) and the Cape Coast Teaching Hospital Ethical Review Committee (CCTHERC/EC/2021/062). Written permission was obtained from each administrative head of the health facilities where the study was conducted. All participants consented after being informed that participation in the survey was voluntary and all aspects of the study, including the objectives and participants' responsibilities and rights in the study, were explained. Participants’ privacy, confidentiality and anonymity were protected.

## 3 Results

### 3.1 Socio-demographic characteristics

A total of 613 health professionals were involved in the study, of whom 306 (49.9%) were female and 307 (50.1%) were male representing the majority. About forty-three percent, 266 (43.4%) of the respondents were below 30 years old, and 248 (40.5%) were between 30 and 39 years old. Regarding educational level, 243 (39.6%) of the health professionals had completed a bachelor’s degree, and 80 (13.1%) had obtained a master’s degree. The majority of the health professionals 302 (49.4%) were single, while 297 (48.5%) were married. More than three-quarters of the staff 487 (78.0%) worked full-time, while the remaining 42 (6.9%) were part-timers and 93 (15.2%) were temporary or casual workers. The majority of staff 282 (46.0%) had spent between 1 and 5 years working in the current facilities. The majority of the staff 503 (82.1%) had access to computers at their workplace and 359 (58.6%) used the computers several times per day, while 72 (11.8%) used them a few times a week to perform their routine duties. The majority of the health professionals were either very comfortable 320 (52.2%) or comfortable 259 (42.3%) using computers. Only 34 (5.5%) were not comfortable using computers (Table 2).

**Table 2:** Demographic characteristics of health professionals from the six selected study sites in Ghana.

Characteristics	Number of Respondents [N = 613]	Percent
Age group (years)		
Below 30	266	43.4
30–39	248	40.5
40–49	77	12.6
50–59	21	3.4
60 plus	1	0.2
Gender		
Male	307	50.1
Female	306	49.9
Educational level		
Masters	80	13.1
BSc	243	39.6
Higher National Diploma	28	4.6
Diploma	184	30.0
Senior Secondary School	9	1.5
Others	69	11.3
Marital status		
Married	297	48.5
Single	302	49.4
Divorced	7	1.1
Widow/Widower	6	1.0
Profession		
Physicians/Doctors	122	20.0
Nurses/Midwives	336	55.0
Pharmacy Technicians	34	5.6
Dietetics/Nutritionist	10	1.6
Radiologist	13	2.1
Physiotherapist	6	1.0
Others	90	14.7
Employment status		
Full-time	487	78.0
Part-time	42	6.9
Temporal/Casual	93	15.2
Total years of service		
Less than 1	168	27.4
1–5	282	46.0
6–10	97	15.8
11–15	31	5.1
15 plus	35	5.7
Total years of practice		
Less than 1	209	34.1
1–5	310	50.6
6–10	63	10.3
11–15	10	1.6
15 plus	21	3.4
Comfortable using Computers		
Very comfortable	320	52.2
Comfortable	259	42.3
Not comfortable	34	5.5
Access to computers at workplace		
Yes	503	82.1
No	110	17.9
Access to computers at home		
Yes	473	77.2
No	140	22.8
How often do you use computers at workplace?		
Not at all	99	16.0
About once each month	9	1.5
A few times a month	18	2.9
About once each week	14	2.3
A few times a week	72	11.8
5 to 6 times a week	20	3.3
Once a day	21	3.4
Several times a day	359	58.6
Others	2	0.3
Do you share computers with colleagues?		
Yes	479	78.1
No	134	21.9

Data are presented as frequencies and percentages; N - total number of participants.

### 3.2 Descriptive statistics, scale and Item reliability test

The overall mean analysis of health professionals' perceived telemedicine readiness with their subscales is presented in Table 3. The overall mean score for the facility’s core readiness, staff engagement readiness, staff knowledge and attitude readiness, and health professionals' readiness was above 3. This shows that a favourable mean score above 3 (neutral) indicates health professionals agree that the measures outlined support their readiness to use telemedicine to provide services to clients. The Cronbach’s alpha item reliability test showed that the scale is internally reliable [42]. For each study construct, Cronbach’s alpha coefficient ranges from 0.78 to 0.87. The Content Validity Index (CVI) was 87.1%, and the Content Validity Ratio (CVR) was 93.5%, respectively.

**Table 3:** Descriptive statistics, scale and item reliability test of study constructs.

Construct	No of Items	Mean response	Standard Deviation	Mean Values under 95% Confidence Interval	Cronbach’s Alpha
Core Readiness (CR)	11	3.46	0.50	[3.42–3.50]	0.83
Engagement Readiness (ER)	6	3.40	0.55	[3.35–3.44]	0.78
Knowledge and Attitude Readiness (KAR)	10	3.69	0.56	[3.64–3.73]	0.82
Health Professionals Readiness (HPR)	4	3.63	0.72	[3.58–3.69]	0.87

The Kaiser–Meyer–Olkin measure of sampling adequacy = 0.910.

Bartlett's test of sphericity, chi-square = 8839.944, Degree of freedom (DF) = 465, significance <0.0001.

### 3.3 Relationship between core, engagement, knowledge and attitude and health professionals’ readiness

**Fig. 1:**
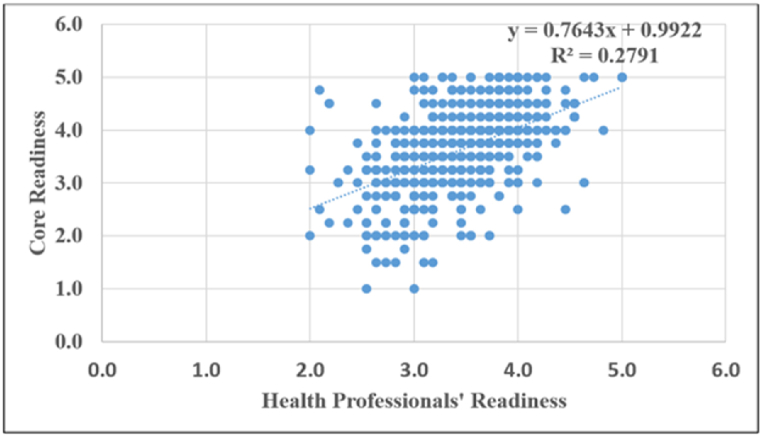
Scatterplot showing the relationship between facility core readiness and health prefessionals’readiness.spearman Rank correlation (r) between facilities core readiness and health professionals readiness [r = 0.5484; p < 0.0001]. Source: Authors' analysis

**Fig. 2:**
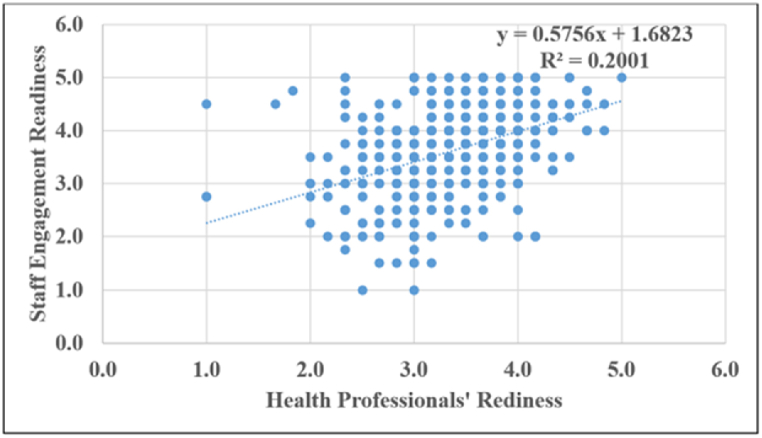
Scatterplot showing the relationship between staff engagement readiness and health prefessionals’readiness.spearman Rank Correlation (r) between staff engagement readiness and health professionals' readiness [r = 0.4882; p < 0.0001]. Source: Authors' analysis

**Fig. 3:**
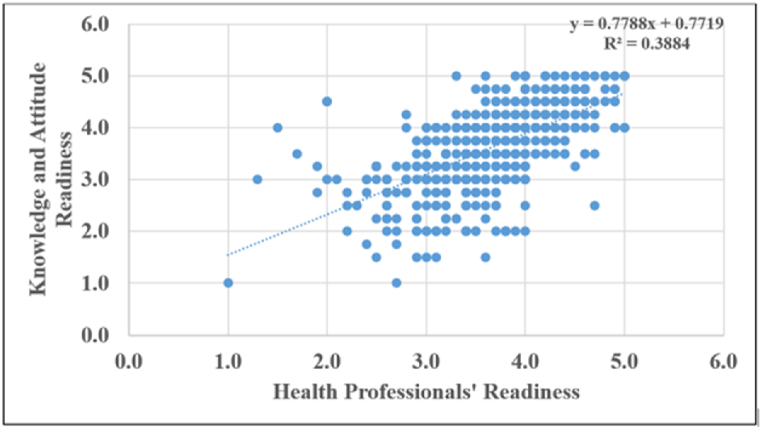
Scatterplot showing the relationship between staff knowledge and attitude readiness and health prefessionals’readiness.spearman Rank correlation (r) between knowledge and attitude readiness and health professionals' readiness [r = 0.6701; p < 0.0001]. Source: Authors' analysis

Fig. 1, Fig. 2, Fig. 3 show spearman’s rank correlation coefficient (r) between core, engagement, knowledge and attitude, and health professionals' readiness. Analysis revealed a statistically significant positive relationship between health facilities' core readiness and health professionals' readiness with r = 0.5484 and p-value<0.0001 (Fig. 1). The study further revealed strong evidence of a statistically significant relationship between staff engagement readiness and health professionals' readiness (r = 0.4882; p < 0.0001) (Fig. 2). Furthermore, staff knowledge and attitude readiness also showed strong evidence of a statistically significant positive relationship with health professionals' readiness (r = 0.6701; p < 0.0001) (Fig. 3).

### 3.4 Exploring factors influencing health professionals’ readiness to implement and use telemedicine

Table 4 shows the unadjusted logistics regression of factors influencing health professionals' readiness towards telemedicine implementation in selected health facilities in Ghana. The outcome from the unadjusted model showed that educational level, total years of service, comfortable using computers, access to computers at the workplace, and frequency of using computers at work had a statistically significant relationship with health professionals' readiness. The analysis further revealed that facility core readiness, staff engagement readiness, and staff knowledge and attitude readiness also had a statistically significant relationship with health professionals' readiness. Unadjusted logistic regression showed that there was a significant association between healthcare professional readiness and core readiness. The evidence revealed that there was a 0.77 (p < 0.001) mean score increase in health professionals' readiness due to facility core readiness. Further analysis confirmed that there was a significant association between healthcare professional readiness and staff engagement readiness. There was a 0.61 (p < 0.001) increase in the mean score of health professionals' readiness due to staff engagement readiness. Finally, the analysis showed that there was a significant association between healthcare professional readiness, and staff knowledge and attitude readiness. The unadjusted model showed that there was a 0.83 (p < 0.001) mean score increase in health professionals’ readiness due to staff knowledge and attitude readiness (Table 4).

**Table 4:** Exploring factors affecting health professionals’ readiness towards telemedicine implementation in six selected health facilities in Ghana.

Characteristics	Unadjusted		
	Coefficient	95% CI	*p*-value
Age group (years)			
Below 30	ref		
30–39	−0.09	[-0.22–0.04]	0.130
40–49	−0.02	[-0.20–0.17]	0.525
50–59	−0.21	[-0.54–0.11]	0.031
60 plus	0.32	[-1.10–1.74]	0.669
Gender			
Female	ref		
Male	0.06	[-0.05–0.18]	0.273
Educational level			
BSc	ref		
Masters	−0.05	[-0.23–0.13]	0.592
Higher National Diploma	−0.50	[-0.78 to −0.22]	0.001**
Diploma	−0.19	[-0.33 to −0.06]	0.006*
Senior Secondary School	−0.10	[-0.58–0.37]	0.668
Others	0.06	[-0.13–0.25]	0.560
Marital status			
Single	ref		
Married	0.07	[-0.05–0.18]	0.260
Divorced	0.33	[-0.21–0.87]	0.229
Widow/Widower	0.24	[-0.35–0.82]	0.425
Profession			
Nurses/Midwives	ref		
Physicians/Doctors	0.10	[-0.05–0.25]	0.212
Pharmacy Technicians	0.14	[-0.11–0.40]	0.278
Dietetics/Nutritionist	−0.23	[-0.68–0.23]	0.331
Radiologist	0.01	[-0.39–0.42]	0.945
Physiotherapist	0.23	[-0.35–0.82]	0.437
Employment status			
Full-time	ref		
Part-time	−0.06	[-0.29–0.16]	0.584
Temporal/Casual	−0.03	[-0.19–0.13]	0.730
Total years of service			
1–5	ref		
Less than 1	−0.05	[-0.19–0.09]	0.482
6–10	−0.01	[-0.16–0.17]	0.929
11–15	−0.37	[-0.63 to −0.10]	0.007*
15 plus	−0.17	[-0.42–0.09]	0.199
Total years of practice			
1–5	ref		
Less than 1	0.08	[-0.05–0.21]	0.218
6–10	−0.12	[-0.31–0.08]	0.248
11–15	0.13	[-0.32–0.59]	0.562
15 plus	−0.01	[-0.33–0.31]	0.960
Comfortable using Computers			
Very comfortable	ref		
Comfortable	−0.10	[-0.22–0.01]	0.085
Not comfortable	−0.44	[-0.70 to −0.19]	0.001*
Access to computers at work place			
Yes	ref		
No	−0.17	[-0.32 to −0.02]	0.025*
Access to computers at home			
Yes	ref		
No	−0.11	[-0.24–0.03]	0.124
Frequency of use of computers at work place			
Several times a day	ref		
Not at all	−0.31	[-0.47 to −0.16]	<0.001**
About once each month	−0.54	[-1.00 to −0.08]	0.021*
A few times a month	0.01	[-0.33–0.33]	0.990
About once each week	−0.46	[-0.83 to −0.09]	0.015*
A few times a week	−0.56	[-0.73 to −0.38]	<0.001**
5 to 6 times a week	−0.12	[-0.43–0.20]	0.468
Once a day	0.13	[-0.18–0.43]	0.417
Others	−0.02	[-0.98–0.95]	0.974
Do you share computers with colleagues			
Yes	ref		
No	−0.06	[-0.20–0.07]	0.361
Core Readiness (CR)	0.77	[0.67–0.87]	<0.001**
Engagement Readiness (ER)	0.61	[0.52–0.70]	<0.001**
Knowledge and Attitude Readiness (KAR)	0.83	[0.75–0.90]	<0.001**

Notes: *p < 0.05, **p < 0.001 were considered statistically significant.

CI – confidence interval; ref-reference group.

## 4 Discussion

This study assessed the readiness of health professionals in six selected health facilities towards the implementation of telemedicine. Most of the health professionals in this study had access to computers, one of the principal technological tools involved in the practice of telemedicine. This is a positive finding, since lack of access to computers, especially in many LMICs, has been reported as a major barrier to the adoption of similar technologies in workplaces [26,43]. However, we need to be cautious not to overemphasise this point, since other technological gaps may exist despite the positive correlation [30,44].

The current study also revealed that the majority of health professionals were comfortable or very comfortable using computers. This is not surprising since the majority of the respondents in the study were in the younger age groups. Studies [38,43] have shown that computer or technology acceptance is positively correlated with younger age groups. This is because younger people have a natural tendency and interest, to adopt new technology as compared to their older counterparts, and therefore show better readiness for new technologies.

Another important finding is that most health professionals use computers often to perform routine tasks, an indication that they have the skills and positive attitude towards the use of computers. The current study demonstrated that access to computers at the workplace and the frequency of use of these computers at the workplace were statistically significantly associated with health professional readiness. Having prior knowledge of computers breeds a positive attitude towards their use [43,45]. This puts health professionals in a ready position to transition to telemedicine. Studies [31,38] have shown that awareness and use of similar technology systems may be translated into core clinical and learning readiness. It is encouraging that health professionals who were computer literate, use computers and/or had computers at work were likely to be eager to use similar technology. A growing body of literature has shown that poor computer skills or an unwillingness to use computers are related to poor readiness [31,38]. The current study, therefore, showed that health professionals were ready to adopt telemedicine.

In our assessment the overall mean scores for health facility’s core readiness, engagement readiness, knowledge and attitude readiness, and health professionals' readiness towards implementation of telemedicine were above 3.4, an indication that there were favourable responses from health professionals that the selected health facilities had some measures put in place for the use of telemedicine to provide health services to their clients. In this current study, facility core readiness has shown a statistically significant relationship with health professionals' readiness. This shows that once healthcare providers can put in place measures to facilitate telehealth, health professionals are likely to respond positively knowing that these tools would facilitate their ability to provide quality and safe healthcare to their clients remotely at a lower cost. These studies [27,43,[46], [47], [48]] in LMICs have shown that health professionals are ready to embrace new technologies with the support of their employees. The study showed that health professionals believe that there is enough plan in place supported by adequate technical infrastructure to implement telemedicine. This is evidenced by the 0.76 mean score increase in health professionals' readiness due to facility core readiness in the unadjusted model. The availability of the technical infrastructure in the facility can change the poor perception health professionals might have, building their confidence that when they transition to telemedicine it will be successful. This result is comparable to Ref. [38], where the presence of good technical equipment, access to computers, and frequent use resulted in an increased readiness of the health professionals.

The study further revealed that facility engagement readiness has a significant relationship with health professionals’ readiness. This shows that healthcare providers can engage and create awareness among staff on the role of telemedicine in providing remote quality health care to their clients. This also echoes the fact that the involvement of stakeholders in planning and decision-making is an important component of technology adoption which can influence their belief in success and must therefore not be ignored.

Again, the study also showed that having the right knowledge and attitude is an important factor that can influence a change of perception. When health professionals have higher educational levels and are comfortable using computers at work, they turn out to be more willing to adopt new technologies. This was confirmed in our study where the level of education of health professionals and health professionals' knowledge and attitude readiness were statistically significantly associated with health professionals’ readiness. On the contrary, poor computer skills of health professionals have been associated with poor readiness of health professionals for technological systems [38,43,45,48]. This result also echoes the need for adequate training so that health professionals gain the right knowledge and attitude. Several studies have shown that many eHealth technologies failed to take up because health professionals were not interested in change and want to keep the status quo [9,10,49,50].

## 5 Limitations

One limitation of the study was the use of the convenience sampling method in the selection of the respondents. This may have introduced a selection bias and limited the generalizability of the findings. Another limitation is that respondents self-completed the questionnaires, and their responses may be based on perceptions [51]. Future studies must endeavour to include semi-structured interviews to explore for details or validate the findings.

## 6 Conclusion

The majority of health professionals in the selected health facilities are ready to adopt and use telemedicine. Telemedicine implementation success and use in LMICs hinges on the availability of several factors, such as policy framework, trust and awareness of the health professionals in the technology and reliable ICT infrastructure. Many eHealth projects in LMICs are largely initiated and funded by central governments and are often left unsustained when funding withers off. We highlighted the factors which can help sustain the implementation and use of telemedicine. Awareness can be raised through the training and education of health professionals. This will encourage health facilities and professionals to prioritise their needs and prepare towards telemedicine adoption.

## Author contribution statement

Nathan Kumasenu Mensah; Godwin Adzakpah: Conceived and designed the experiments; Performed the experiments; Analyzed and interpreted the data; Contributed reagents, materials, analysis tools or data; Wrote the paper. Richard Okyere Boadu; Jonathan Kissi; Obed Uwumbornyi Lasim; Martha Khainde Oyenike; Abigail Bart-Plange: Performed the experiments; Analyzed and interpreted the data; Contributed reagents, materials, analysis tools or data; Wrote the paper. Maxwell Ayindenaba Dalaba; Felix Sukums: Analyzed and interpreted the data; Contributed reagents, materials, analysis tools or data; Wrote the paper.

## Funding statement

This research did not receive any specific grant from funding agencies in the public, commercial, or not-for-profit sectors.

## Data availability statement

Data will be made available on request.
